# Zona incerta as a therapeutic target in Parkinson’s disease

**DOI:** 10.1007/s00415-019-09486-8

**Published:** 2019-08-02

**Authors:** Krystyna Ossowska

**Affiliations:** Department of Neuropsychopharmacology, Maj Institute of Pharmacology, Polish Academy of Sciences, 12 Smętna St, 31-343 Kraków, Poland

**Keywords:** Deep-brain stimulation, Zona incerta, Parkinson’s disease, Animal studies, Anatomical studies, Clinical studies

## Abstract

The zona incerta has recently become an important target for deep-brain stimulation (DBS) in Parkinson’s disease (PD). The present review summarizes clinical, animal and anatomical data which have indicated an important role of this structure in PD, and discusses potential mechanisms involved in therapeutic effects of DBS. Animal studies have suggested initially some role of neurons as well as GABAergic and glutamatergic receptors of the zona incerta in locomotion and generation of PD signs. Anatomical data have indicated that thanks to its multiple interconnections with the basal ganglia, thalamus, cerebral cortex, brainstem, spinal cord and cerebellum, the zona incerta is an important link in a neuronal chain transmitting impulses involved in PD pathology. Finally, clinical studies have shown that DBS of this structure alleviates parkinsonian bradykinesia, muscle rigidity and tremor. DBS of caudal zona incerta seemed to be the most effective therapeutic intervention, especially with regard to reduction of PD tremor as well as other forms of tremor.

## Introduction

It is generally accepted that motor signs of Parkinson’s disease (PD) akinesia, bradykinesia, muscle rigidity and tremor are related to the loss of dopamine in the striatum (putamen and caudate nucleus) due to degeneration of the dopaminergic nigrostriatal pathway arising from the substantia nigra pars compacta (SNc) [1]. In line with this view, a dopamine precursor, levodopa, became a gold standard of antiparkinsonian therapy [2, 3]. However, after a few years of treatment with this drug extremely troublesome side effects, i.e. dyskinesias and on–off phenomenon appear. Introduction of deep-brain stimulation (DBS) to the therapy of PD patients with advanced disease demonstrating strong uncontrollable levodopa-induced motor complications allowed for alleviation of parkinsonian signs as well as reduction of levodopa doses and side effects [3]. DBS of a few brain regions, i.e. ventral intermediate nucleus (Vim) [a synonym of posterior portion of the ventrolateral nucleus (VLp)] of the thalamus, ventrolateral region of the internal segment of the globus pallidus (GPi), the subthalamic nucleus (STN) and tegmental pedunculopontine nucleus (PPN) have been found to be beneficial in PD [4–9]. Among these structures, the STN is currently the most frequently chosen target for this procedure [4, 6, 9]. However, a number of recent clinical data have indicated that the zona incerta (ZI) and white matter located in the vicinity of the STN may be an alternative or concomitant target for DBS [10].

## Cortico-basal ganglia-thalamo-cortical network and DBS of the STN

It is generally accepted that voluntary movements are initiated in the motor cortex which sends projections to the brainstem, spinal cord and subcortical targets (the basal ganglia). According to the classic model, the role of the basal ganglia output pathways to the thalamus is to facilitate or suppress movements by controlling information transmitted via the thalamocortical projection to the cortex. The basal ganglia-induced inhibition of the thalamic neurons has been suggested to be related to reduction of movements, while their disinhibition—with movement activation [11].

**Fig. 1:**
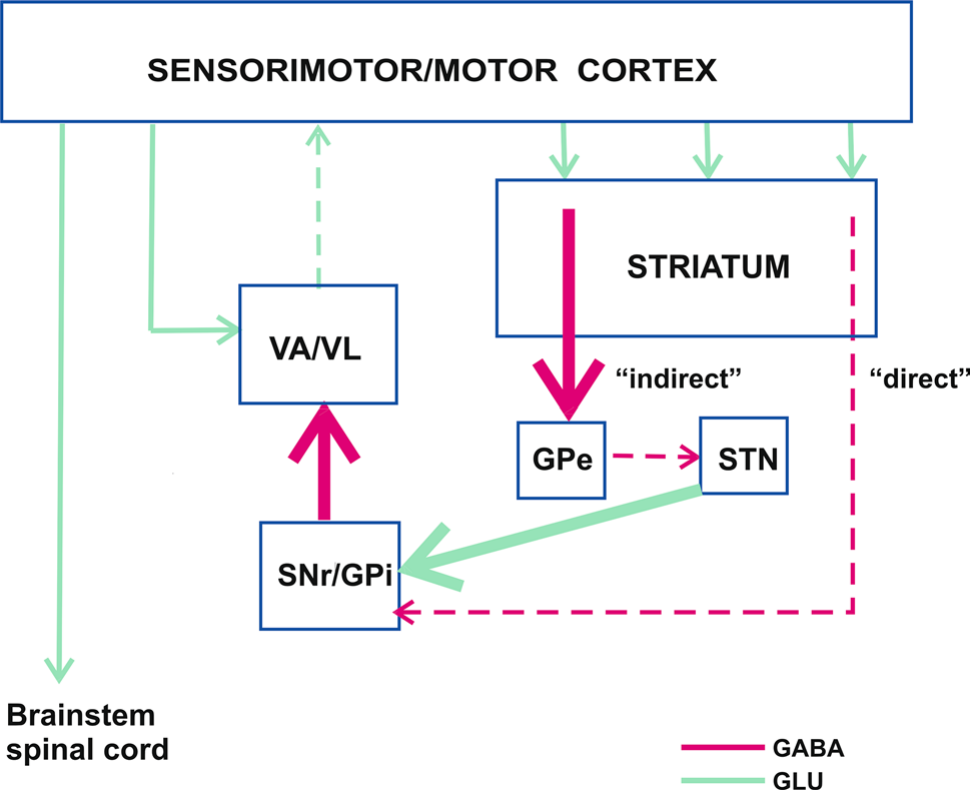
A “classic” model of neuronal activity in the cortico-basal ganglia-thalamo-cortical circuit in Parkinson’s disease (PD) according to DeLong [13] (modified). Bold arrows indicate activated neuronal pathways, dashed arrows indicate inhibited neuronal pathways. Due to dopaminergic deficiency in PD, the “indirect” GABAergic pathway from the putamen (a part of the striatum) to GPe is disinhibited which results in inhibition of the GABAergic pathway from the GPe to the STN, and disinhibition of the glutamatergic route projecting from the STN to the SNr/GPi. On the other hand, loss of dopamine leads to decreased activation of the “direct” GABAergic pathway projecting from the putamen to the SNr/GPi. Finally, glutamatergic activation of the SNr/GPi prevails over GABAergic inhibition of these structures, which leads to activation of their output GABAergic pathways going to the VA/VL nuclei of the thalamus. As a result, glutamatergic efferents of these thalamic nuclei to the sensorimotor/motor cortex are inhibited. Brain structures: *GPe* the external segment of the globus pallidus, *GPi* the internal segment of the globus pallidus, *SNr* the substantia nigra pars reticulata, *STN* the subthalamic nucleus, *VA/VL* ventroanterior/ventrolateral nuclei of the thalamus. Neuronal pathways: *GABA* GABAergic pathway, *GLU* glutamatergic pathway

Degeneration of dopaminergic nigrostriatal pathway, arising from the SNc leads to a number of functional alterations in structures belonging to the above cortico-basal ganglia-thalamo-cortical neuronal loop which underlie motor signs of PD [6, 11–14] (Fig. 1). Its first link, i.e. the corticostriatal glutamatergic projection in humans starts from the precentral motor region (primary motor cortex, premotor cortex and supplementary cortex) and postcentral somatosensory cortex, and terminates largely in the putamen where it switches to medium-spiny GABAergic neurons which form two striatal efferents: the “direct” pathway leading to the substantia nigra pars reticulata (SNr) and ventrolateral region of the GPi, and the “indirect” pathway going to the external segment of the globus pallidus (GPe). The next link of the “indirect” pathway, i.e. the GABAergic pallidosubthalamic projection inhibits glutamatergic neurons of the STN which form subthalamonigral (leading to the SNr) and subthalamopallidal (leading to the GPi) pathways. Finally, glutamate released from terminals of these pathways activates GABAergic neurons of the SNr and GPi which send their axons to the motor thalamus, i.e. to the ventroanterior (VA) nucleus and anterior portion of the ventrolateral nucleus (VL), respectively. It has been suggested that the pallidothalamic pathway is involved in sequencing and execution of movements, while the nigrothalamic projection contributes to planning of movements. GABAergic basal ganglia-thalamic pathways, in turn, inhibit glutamatergic thalamocortical projections which close the cortico-basal ganglia-thalamo-cortical neuronal loop [6, 11–14].

Several lines of evidence have indicated that the loss of dopamine in PD results in an imbalance between the “indirect” and “direct” pathways, i.e. the “indirect” pathway is released from inhibition and the activation of the “direct” pathway is reduced. That leads first to disinhibition of the STN neurons, then to activation of GABAergic basal ganglia output pathways which finally results in hyperinhibition of thalamocortical neurons [6, 11–14] (Fig. 1).

It must be stressed, however, that this model of PD-related neuronal network is, in fact, much more complicated and includes additionally some other connections, i.e. GABAergic projections from the SNr to the superior colliculus, PPN and intralaminar thalamic nuclei, and from the GPe to SNr and GPi, as well as glutamatergic projections from the cerebral cortex to the STN and thalamic nuclei, from the STN to the GPe, and others [6, 11, 13, 15]. Moreover, this model is not sufficient to explain pathomechanisms of PD tremor which seem to be distinct from those underlying bradykinesia and rigidity. According to recent studies, the tremor-related mechanisms include increased interaction between the cerebello-thalamo-cortical network and the basal ganglia [6, 16, 17]. In fact, the subcortical nuclei of the cerebellum communicate transsynaptically with the putamen and GPe via motor and intralaminar nuclei of the thalamus [18, 19] while the STN sends di-synaptic connections to the cerebellar cortex [20, 21]. Although a “pacemaker” of parkinsonian tremor has not been identified, yet, it has been suggested that “the basal ganglia network triggers the onset of tremor and the cerebellar network is responsible for maintaining the tremor rhythm and its amplification” [16, 17].

In line with the above model, pathological excitation of the STN neurons in PD patients or parkinsonian monkeys, characterized by irregular and bursty pattern or periodic oscillatory bursts has been suggested to be associated to akinesia/muscle rigidity and parkinsonian tremor, respectively [22–24]. This view was supported by animal studies which showed that lesions of this region in parkinsonian monkeys [25–27] and accidental hematoma in PD patient dramatically reduced akinesia, tremor and muscle rigidity [28]. These studies inspired researchers to introduce DBS of the STN, first in parkinsonian monkeys [29], then in PD patients [30]. Spectacular therapeutic results of these studies (reduction of PD signs and lack of dyskinesias) [29, 30] initiated a new age of surgical interventions in PD and DBS of the STN was approved by the United States Food & Drug Administration (FDA) in 2001 [4]. Since that time thousands of DBS of the STN carried out all over the world have supported a significant long-term therapeutic efficacy of this procedure.

The STN is functionally and anatomically divided into three subdivisions: motor (dorsolateral), associative (ventromedial) and limbic (medial) with separate input and output pathways, although, it remains unclear to what extent these regions overlap [31, 32]. According to this functional division, the dorsolateral part of this structure is usually chosen for DBS in PD which results in reduction of bradykinesia, muscle rigidity and tremor [10, 33].

However, according to some studies DBS of the anterior dorsolateral border of the STN [34, 35] or the interface between this region and ZI and thalamic fasciculus located dorsally to the STN [36] are equally [34, 36] or even more effective than DBS inside the STN [35].

## Active target for DBS outside the STN: the ZI

The first evidence of the role of the ZI in PD came from the paper of Mundinger [37] who observed that high-frequency coagulation of a region which included the caudal ZI, H1/H2 Forel’s fields and partly prelemniscal radiation and nucleus ruber was highly effective in ameliorating muscle rigidity and tremor in PD patients. Similarly, Patel and coworkers [38] reported a strong antiparkinsonian, mainly tremorolytic, effect as a result of a lesion of the ZI/H2 region. Moreover, patients with a selective dorsolateral subthalamotomy demonstrated less clinical benefit than those with a combined lesion which included both the STN and the dorsally adjoining ZI/H2 region [38].

Research which compared clinical potency of DBS at different anatomical electrode locations in the STN and its surroundings has shown that stimulation of the region localized dorsally to this structure which included the ZI and/or H1/H2 Forel’s fields was weaker, equal or stronger than that of the STN.

While Herzog et al. [34] reported that DBS of the ZI and H1/H2 (located > 1.5 mm dorsally to the STN) was beneficial, its clinical effect in comparison to the STN appeared only suboptimal. Similarly, Welter et al. [39] claimed that “the best motor outcome was obtained when stimulating contacts were located within the STN as compared with the ZI”. Moreover, de Chazeron et al. [40] found that when both DBS contacts were located inside the STN or one inside the STN and the second one at its superior border, the motor improvement was better than when both contacts were localized outside, viz. superolaterally to this structure, presumably in the ZI. According to Yokoyama et al., [41] stimulation delivered dorsally to the STN to the rostral ZI/ pallidofugal fibres was as effective as stimulation inside the STN. Similarly, Henderson et al. [42] observed that when electrodes were positioned bilaterally: one at the border of the thalamus and ZI, and the second inside the STN, reduction of tremor, muscle rigidity and bradykinesia induced by DBS was similar on both sides of the body.

Voges and coworkers [43] and Lanotte et al. [33] were the first who claimed advantage of DBS of regions located dorsally to the STN. Voges et al. [43] observed that the relative amelioration of contralateral motor signs per energy unit was the highest after DBS of the region containing Forel’s fields and rostral part of the ZI. Lanotte et al. [33] found that 50% of the most effective electrode contacts (with respect to a decrease in the muscle rigidity of the wrist) were located 0.5 mm above the upper limit of the STN.

However, in the light of proximity of the above-mentioned stimulated regions of ZI and Forel’s fields to the STN and considering difficulties in determining the extent of current spread from electrodes, some doubts were raised as to their real contribution to DBS therapeutic effects. Since according to Pollak et al. [44], DBS is capable of affecting a tissue area of 2–3 mm in diameter, Yokoyama et al. [41] and Lanotte et al. [33] have suggested that the clinical effects described by them and induced by stimulation of areas located outside the STN might result from current spread to the latter structure.

Addressing this problem, Maks and coworkers [45] applied a method allowing for theoretical prediction and visualization of the volume of tissue activated (VTA) by clinically defined therapeutic parameters of STN stimulation with monopolar electrodes in 10 PD patients. In their work, the anatomical electrode localization was determined by neuroimaging and neurophysiological recordings. According to their calculations, which were based on neurostimulation parameters, capacitance and impedance of the electrode–tissue interface and electrical properties of tissue surrounding electrodes, the VTA was equal to 71 mm^3^ and was smaller than that of the whole STN (ca. 200 mm^3^) [45]. Using this method, the authors found that patients who had more than half of the VTA outside the STN (dorsally to this structure) demonstrated better therapeutic outcome than those who had more than a half of the VTA inside the STN [45].

While in the aforementioned studies therapeutic effects of DBS of more rostral regions of the ZI (and/or Forel’s fields) were examined, Kitagawa et al. [46] proposed that the region of caudal ZI and prelemniscal radiation located posteromedially to the STN can be considered another surgical target, especially for the tremor-dominant PD. They found that stimulation of this region significantly reduced contralateral muscle rigidity, tremor and akinesia, and improved handwriting, posture and gait. Plaha et al. [47] compared effects of stimulation of caudal ZI (located posteromedially and posterodorsally to the STN) to those of the dorsal half of the STN, and to the region located dorsomedially and medially to the STN, which comprised pallidofugal fibres and rostral ZI. Their caudal ZI targets were located more laterally than those investigated by Kitagawa et al. [46]. They found that although DBS of all regions strongly reduced contralateral PD signs (tremor, muscle rigidity, bradykinesia and timed hand movements), stimulation of the caudal ZI was the most effective. Moreover, stimulation of the rostral ZI induced side effects in some patients, i.e. speech deterioration and a sense of disequilibrium during walking [47], as well as irritability, psychomotor agitation and severe progressive insomnia [48]. The former disturbances were not present after stimulation of the caudal ZI [47]. Two years later Plaha and coworkers reported a dramatic mitigation of resting (94.8%) and postural (88.2%) tremor in PD patients as a result of bilateral stimulation of the caudal zone of the ZI [49]. Finally, Blomstedt and coworkers [50] chose this part of the ZI for DBS in 19 PD patients. The stimulated region was located between the red nucleus and STN, slightly posteromedially to the posterior tail of the latter structure. In an agreement with previous studies [47, 49], DBS of this region induced a profound effect on tremor, had less influence on akinesia and was free of negative influence on speech [50]. Special involvement of the caudal ZI and prelemniscal radiation (located posteromedially to the posterodorsal STN) in tremor, in general, was proven also by findings that DBS of this region had strong effect on PD voice tremor, proximal, distal and axial essential tremor, postural and intention component of multiple sclerosis tremor, Holmes tremor (resting, postural and intention) and dystonic tremor [49, 51–54]. When therapeutic effectiveness of DBS in essential tremor was compared in relation to the location of stimulated fields, the best region seemed to incorporate the superior part of the cerebellothalamic fasciculus and caudal ZI [52].

Besides motor signs, several non-motor disturbances, e.g. depression, apathy, decline in cognitive functions, hyposmia, sleep disorders as well as autonomic dysfunctions, are commonly observed in PD patients [55]. The influence of DBS of different brain structures on these disorders has been examined less frequently than that on motor signs. In general, DBS has been considered to have a positive impact on them either due to its direct action or because dopaminergic therapy could be reduced [55]. However, a number of non-motor adverse effects after DBS have also been reported [55]. With regard to the DBS of the ZI, only scarce and inconsistent data on non-motor signs are available.

Early case reports indicated that although DBS of the ZI/Forel’s fields in two PD patients (one with a history of previous depression) did not induce chronic depression, it evoked acute depressive/dysphoric mood states which were time-locked to the stimulation [56, 57].

De Chazeron and coworkers [40] did not find any significant differences between depression scores pre- and post-operation (3 and 6 months) in 18 patients treated with DBS of the ZI. However, Burrows and coworkers [58] reported, on the basis of observation of a small group of 11 PD patients who underwent initially DBS of the STN, that changing the stimulation contact to the ZI or to the area located near the latter structure decreased subjective anxiety, depression as well as mild and extreme fear, while worsened recognition of mild sadness. Moreover, Welter et al. [39] found that DBS of the STN region (which in some cases included the ZI/Forel’s field area) improved mood status in 26 of 45 patients who showed symptoms of depression before surgery, while worsened it in 27 initially non-depressive patients, within 1 year after the operation. Although it was not specified which area (STN vs*.* ZI/Forel’s fields) was stimulated in individual patients, postoperative depression was found to be related either to preexisting depressive signs or lower pre- and postoperative cognitive performance but not to contact locations [39].

Apathy, a common clinical feature of PD [59] seems to be worsened by DBS of the STN region [60, 61]. It has been suggested that both the surgery target and the reduction of dopaminergic medication are involved in aggravation of the apathetic condition after STN-DBS [60, 61]. However, apathy which was described to develop in one PD patient who had stimulating contact in the ZI (located dorsally to the STN) was insensitive to the treatment with the agonist of dopaminergic receptors ropinirole [60].

With regard to non-psychiatric signs, stimulation of the ZI was found to increase appetite [40], and stimulation of the region located ventrally to the motor thalamus (ZI and Forel’s field’s) was reported to induce weak olfactory deficits [62]. However, the latter deficit was only subclinical, because it could be found in specific tests but was not perceived by patients [62]. Moreover, heat sensation, sweating and oculomotor disturbances were sometimes seen during stimulation of the ZI [56, 57, 63, 64].

## Anatomy of the ZI: chemoarchitecture and connections

Neuronal mechanisms underlying therapeutic efficiency of ZI DBS is unknown and may be complex because of differential neuroarchitecture of this structure and multiplicity of its connections.

**Fig. 2:**
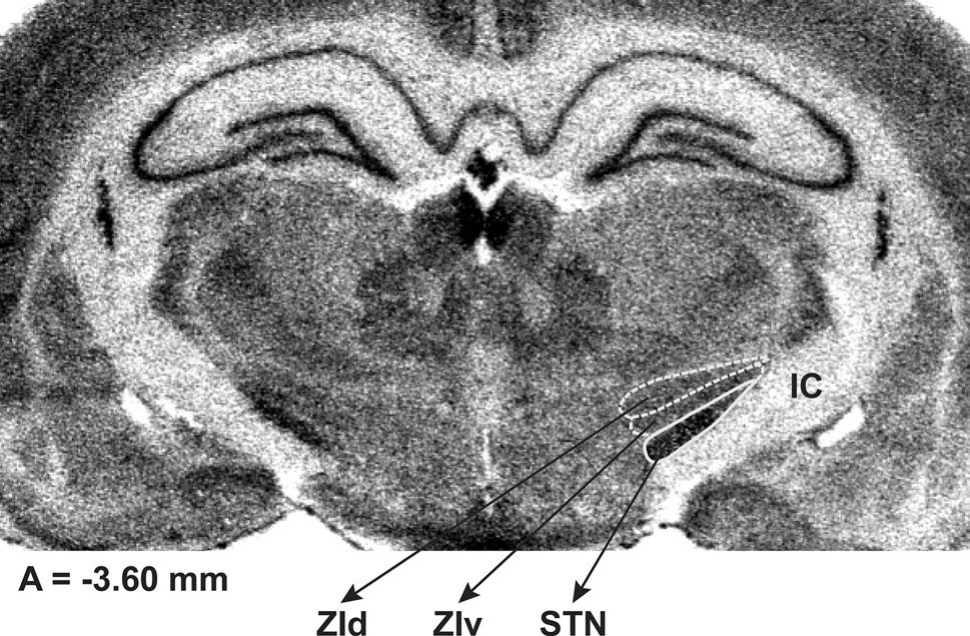
An autoradiogram of mRNA coding for COI (cytochrome oxidase subunit I) in a coronal section of the rat brain, at the level of *A* = − 3.60 mm from bregma, according to the Paxinos and Watson [67]. Borders of ZId, ZIv and STN are marked by white outlines. *IC* internal capsule, *STN* subthalamic nucleus, *ZId* dorsal subdivision of the zona incerta, *ZIv* ventral subdivision of the zona incerta. The autoradiogram has been generously delivered by prof. J. Wardas and Dr. B. Kosmowska (unpublished)

The most knowledge about the ZI anatomy and physiology comes from studies in rodents, however, their results only partly can be compared with those obtained in some other species and humans [65, 66]. The ZI in rats extends from rostral regions of the thalamus to the rostral pole of the red nucleus [66, 67] and in humans from the rostral regions of the thalamus to the level of the rostral pole of the medial geniculate nucleus [66]. The ZI has been divided in rats into 4 main sectors: rostral, dorsal, ventral and caudal [66, 67], although borders between them are not clear [68]. Rostral ZI lies ventrally to the Vm thalamic nucleus [an equivalent of the medial portion of the VA [69] or VL [70] thalamic nucleus in primates], dorsomedially to the entopeduncular nucleus (an equivalent of the GPi) and dorsally to the lateral hypothalamus [67]. Dorsal and ventral sectors in rats are located in the central part of the ZI which lies dorsomedially to the STN and cerebral peduncle, dorsally to the lateral hypothalamus and ventrally to the Vm/ventroposterior thalamus [67] (Fig. 2). Caudal ZI in rats is located dorsally to the cerebral peduncle and the substantia nigra (pars lateralis) and ventrolaterally to parts of the posterior thalamic group of nuclei and medial lemniscus [66, 67]. The “rostral portion” of the ZI used by neurosurgeons for DBS of the region located dorsally/dorsomedially to the STN seems to correspond to the central region of ZI in rats [47, 66]. The caudal ZI stimulated by neurosurgeons lies between cerebral peduncle and substantia nigra, H1 Forel’s field and ventroposterior thalamic nucleus. From the rostral side, it is limited by the STN and from the caudal side by medial lemniscus [66]. The most posterior part of the caudal zona incerta in humans is not targeted by neurosurgeons [66].

Rostral, dorsal, ventral and caudal sectors of the ZI differ significantly with regard to their chemoarchitecture. In rodents, the rostral sector of the ZI comprises the tyrosine hydroxylase-, somatostatin- and glutamate-immunoreactive neurons [71, 72]. Somatostatin-positive neurons are also present in the lateral edge of both dorsal and ventral ZI [68, 72] whereas tyrosine hydroxylase-positive ones are sparse in the medial parts of these sectors [68, 72]. GAD immunoreactive cells are present within all sectors of the ZI, however, most of them which are also parvalbumin-immunoreactive are found within the ventral sector of the ZI [66, 72-75]. The medial part of the dorsal ZI is also rich in parvalbumin-stained cells and neuropil, but the lateral part is not [66]. In the dorsal sector, NADPH-diaphorase, NOS, calretinin and glutamate-immunoreactive cells are the most abundant [66, 71–73]. In an agreement with the above immunoreactivity of neurons, the majority of fibres arising from the ventral tier of the ZI are GABAergic [68, 76, 77], whereas those extending from the dorsal ZI are mainly glutamatergic [71]. In contrast to the ventral ZI, the caudal ZI in rodents expresses little or no parvalbumin, GAD65/67 and GABA transporter immunoreactivity and includes a smaller number of NADPH-diaphorase-, NOS-, glutamate- and calretinin-positive cells than the dorsal tier of the ZI [66, 71]. Instead, the caudal ZI contains a number of calbindin-positive cells and its caudal pole is strongly positive for acetylcholinesterase both in rodents and primates. On the basis of chemoarchitecture of this structure, Watson et al. [66] have postulated that “the caudal pole of the ZI is sharply different in function to the remainder of the ZI”.

**Fig. 3:**
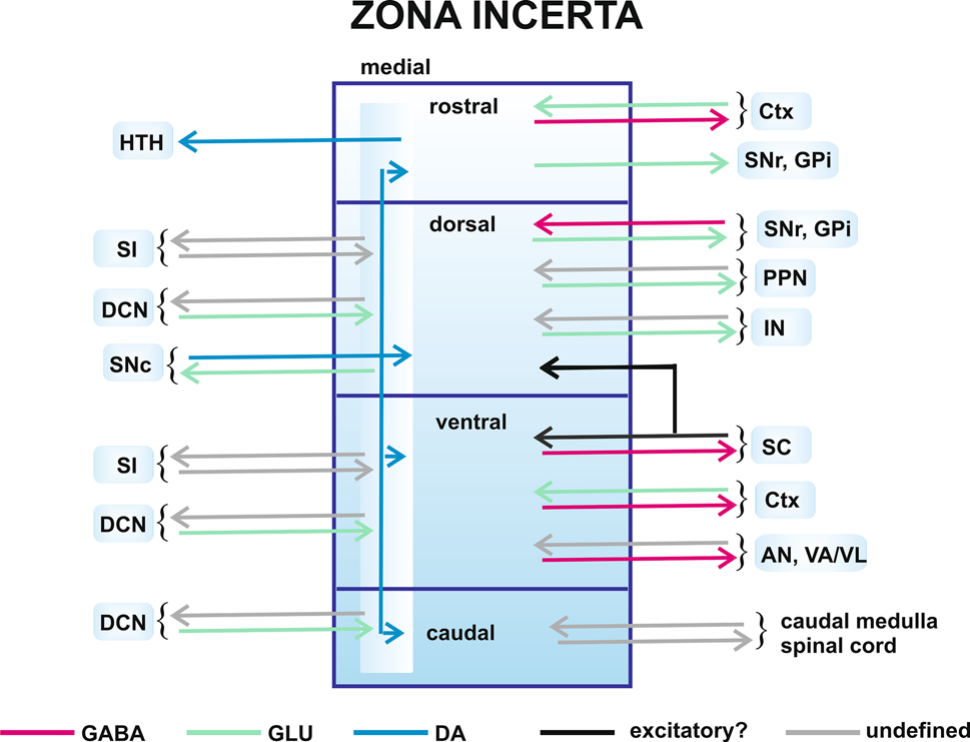
An overview of the main anatomical connections—outputs and input pathways (shown as arrows) of the zona incerta. Brain structures: *AN* association nuclei of the thalamus, *Ctx* cerebral cortex, *DCN* deep cerebellar nuclei, *GPi* the internal segment of the globus pallidus, *HTH* hypothalamus, *IN* intralaminar nuclei of the thalamus, *PPN* pedunculopontine nucleus, *SC* superior colliculus, *SI *substantia innominata, *SNc* substantia nigra pars compacta, *SNr* substantia nigra pars reticulata, *VA/VL* ventroanterior/ventrolateral nuclei of the thalamus. Neuronal pathways: *DA* dopaminergic pathway; excitatory?—putative excitatory pathways, *GABA* GABAergic pathways, *GLU* glutamatergic pathways; undefined—pathways of undefined neurotransmitter. Four subdivisions (rostral, dorsal, ventral and caudal) of the zona incerta are shown. The medial region extends through all the zona incerta subdivisions

Besides the chemoarchitecture diversities, the above regions of the ZI differ with respect to their afferents and efferents (Fig. 3).

### Interconnections with the superior colliculus

The ventral sector of the ZI is the main area which gives rise to the GABAergic pathway to the superior colliculus in rats, cats and primates [78–84]. However, a reciprocal connection from the latter structure terminates in all subdivisions of the ZI, but mostly in its ventral tier in rats [77–79, 85] (Fig. 3) and in the main body, i.e. central ZI in cats and primates [80, 81].

### Interconnections with the basal ganglia and brainstem

Although all sectors of the ZI send fibres to the basal ganglia structures [SNc and SNr, PPN (mainly pars dissipata), GPe and entopeduncular nucleus (GPi)], the incertal projection neurons (mainly glutamatergic) are the most abundant in the dorsal and rostral ZI in rats [71, 79, 82–84] (Fig. 3). Moreover, a few GABAergic neurons in the ventral subdivision of the ZI send their fibres to the SN, PPN and entopeduncular nucleus (GPi) [71, 78]. In contrast to more rostral regions, the caudal ZI has been reported to have only few incerto-basal ganglia neurons [71]. The ZI is traversed by fibres of topographically arranged projection from the SNr to motor higher order thalamic nuclei (VA/VL, intralaminar, and mediodorsal) which is GABAergic [86, 87]. Some of these fibres form terminals in the ZI [87]. Afferents from the SNr and other brainstem nuclei (midbrain reticular nucleus, pontine reticular nucleus, ventral tegmental area, PPN (pars dissipata), dorsal raphe, periaqueductal grey matter) terminate in all sectors of the ZI but mainly in its dorsal part [71, 79] (Fig. 3). On the other hand, dopaminergic projection from the SNc in rats terminates in the medial region of all sectors of the ZI, close to the border of Forel’s fields and lateral hypothalamus in rats [71] (Fig. 3). The ZI receives also the projection from the GPi and is traversed by pallidothalamic fibres (arising from the associative/limbic GPi), which lead to VA/VL and intralaminar thalamic nuclei, and some of them form terminals in the ZI in monkeys, humans and rats [71, 88–91] (Fig. 3).

### Interconnections with the thalamus

All sectors of the ZI send projections to the dorsal thalamus which can be considered as a “gateway” to the neocortex for sensory information. The heaviest projections run to higher order thalamic nuclei: association nuclei (mainly from the ventral ZI) (lateral dorsal nucleus, lateral posterior nucleus and posterior thalamic nucleus) and intralaminar nuclei (mainly from the dorsal ZI) (central lateral nucleus, paracentral, central, medial, parafascicular nucleus (PF), posterior intralaminar nuclei) [68, 76, 77, 82–85, 92] (Fig. 3). Fewer fibres project to the first-order thalamic nuclei (lateral geniculate nucleus, medial geniculate nucleus, ventral geniculate nucleus). Scattered axons of the incerto-thalamic pathways arising from the central region of the ZI were also seen in the VA/VL nuclei in rats [76, 82] (Fig. 3). A vast majority of incerto-thalamic pathway neurons whose terminals innervate the proximal dendrites of relay thalamic neurons have been found to be GABAergic [76]. The ZI receives reciprocal connections from the dorsal thalamic nuclei. Most terminals belonging to fibres arising from intralaminar and association nuclei are present in all sectors of the ZI but predominantly in its dorsal and ventral sector, respectively [92] (Fig. 3). Afferents from primary relay nuclei are only scarce and were found scattered across all sectors of the ZI in rats [92].

### Interconnections with the cerebral cortex

The rostral and central, but not caudal regions of the ZI send projections to the entire cerebral cortex in rats (especially to the frontal and parietal—somatosensory cortex) [75, 93–95] (Fig. 3). These projections are at least partly GABAergic [93]. Reciprocal excitatory projections from layer V of most cortical regions (frontal, cingulate, parietal, forelimb, and occipital) to the ZI have been shown [68, 96–98]. The heaviest projection originates in the cingulate cortex, whereas the weakest in the occipital cortex [97]. Among others, pathways originating in the parietal cortex, cortical representation of forelimbs, and vibrissae motor cortex terminate mainly in the ventral tier of the ZI (Fig. 3) with smaller representation in the dorsal ZI [68, 95, 99]. On the other hand, the cingulate cortex sends heavy projections to the dorsal and rostral sectors of the ZI [68, 97].

### Interconnections with the cerebellum and red nucleus

The ZI (especially its more centrocaudal regions) is traversed by cerebellothalamic fibres arising from the dentate, interposed and fastigial subcortical cerebellar nuclei which terminate in VL nucleus (including its posterior portion), intralaminar and other associative thalamic nuclei in rats, monkeys and humans [88, 90, 100]. Some of these fibres form collaterals which terminate in the ZI [100]. Terminals of the projection from the interposed nucleus in rats are localized in all subregions of the ZI, but mainly medially in dorsal and ventral tiers of the ZI [101] (Fig. 3). Their density is especially prominent in the contralateral ZI thus ipsilateral caudal ZI is devoid of them. There is also a reciprocal small projection from the ZI (mainly from its medial region) to the interposed nucleus (Fig. 3). Although cells of the latter projection were found on both sides of the brain, they tended to be more numerous on the ipsilateral side [101].

There are also strong interconnections between the ZI and the red nucleus [102]. All sectors of the ZI (mainly their medial parts) send projections to the red nucleus, largely to its parvocellular lamina [102]. A reciprocal connection from the latter structure to medial region of each sector of the ZI was also noted [102].

### Interconnections with caudal medulla and spinal cord

More caudal regions of the ZI send projections to the caudal medulla (to the inferior olivary complex, among others) and cervical and thoracic spinal cord in rodents [75, 82–84, 95, 103] (Fig. 3). The spinal cord (mainly its cervical levels) projects, in turn, especially to more caudal regions of the ZI [75, 95]. No interconnections with the spinal cord and the rostral ZI were observed [95].

### Dopaminergic projections from the ZI

The medial region of the rostral ZI (A13 cells) sends dopaminergic projections to the hypothalamus (Fig. 3), horizontal limb of the diagonal band of Broca, central nucleus of the amygdala [104], as well as to the dorsolateral periaqueductal grey and mesencephalic locomotor region (MLR), i.e. PPN and cuneiform nucleus [105]. Dopaminergic component in the pathways extending from the medial ZI to PPN and cuneiform nucleus amounts 21.38 and 30.21%, respectively, in mice [105].

### Other incertal interconnections

The ZI receives also somatosensory input from the trigeminal nuclei. This pathway terminates almost exclusively in the ventral part of the ZI [75, 95], however, a few trigeminal and medial lemniscus terminals can also be observed in the dorsal, rostral and caudal subdivisions of the ZI [75].

The region of the ZI located dorsally to the medial part of the STN contains terminals of the projection arising from the substantia innominata of the subpallidal forebrain which may be formed by collaterals of the subpallidal-PPN pathway [106] (Fig. 3). A reciprocal projection from the ZI to the subpallidal region was also described in the rat [82, 104].

### Intrinsic incerto-incertal connections

A study carried out in monkeys has shown that besides projection (principal) neurons, the ZI contains also interneurons (presumably GABAergic) which make contacts with both principal neurons and other interneurons. That may lead to feedforward inhibition or disinhibition of principal neurons [107]. Moreover, widespread intranuclear recurrent GABAergic connections along the ZI neurons [76, 99], as well as incerto-incertal fibres connecting different sectors of the ZI ipsi- and contralaterally have been reported in rats [68, 82, 108]. For example, neurons of the caudal ZI (similarly to those of other sectors) heavily innervate dorsal, ventral and rostral ZI ipsilaterally and to a lesser extent contralaterally [68, 108]. In general, the strongest contralateral incerto-incertal projections are present between corresponding sectors of this structure [108].

## The role of the ZI in motor behaviour and in models of parkinsonism in animals

In line with variety of its connections, the ZI has been suggested to be involved in different functions, i.e. in the control of arousal states and attention (orienting movements of the eyes and head, and whisking) [68, 78, 81, 95, 99, 109], visceral activities (ingestion, drinking, sexual cycles, cardiovascular activities and temperature control) [68, 110], posture, locomotion and other motor behaviours (see below), and neuropathic pain [96, 111–113]. It has been proposed that the global function of the ZI consists in translation of diverse sensory (exteroceptive and interoceptive) incoming signals to appropriate visceral, arousal, attention and posture-locomotion reactions [68].

The role of the region of ZI in locomotor behaviour was explored in animals already in the 1950s. Grossman [114] found that stimulation of the area which included H1 and H2 Forel’s fields and the medial portion of the ZI elicited walking movements in anaesthetized cats. However, the majority of studies of the ZI have been carried out in rats.

Almost 30 years after Grossman’s discovery [114], Mogenson and coworkers [106] proposed that a part of central region of the ZI located dorsally to the medial edge of the STN in rats belonged to the neuronal network involved in locomotor activity which includes pathways connecting nucleus accumbens with PPN (associated with the MLR) via the subpallidal region and ZI. According to their study, subpallidal neurons which are targets for impulses arising from the nucleus accumbens send projections to PPN through ZI where some fibres form collaterals and terminals. Moreover, procaine administered into the above region of ZI which blocked descending projections, reduced locomotor activity induced by a blockade of GABA_A_ receptors by picrotoxin injected into the subpallidal region [106]. In line with the above suggestion and results of the classic paper by Grossman [114], it has been reported that electrical stimulation of ZI or a site within the field of Forel located dorsomedially to the STN triggered locomotor activity measured in an open field or on a treadmill in rats [115–118]. Moreover, such stimulation induced antidromical response in neurons of the subpallidal region, some of which responded also to stimulation of the PPN [116].

The above studies which used electrical stimulation or procaine-induced blockade of impulse flow were not able to differentiate between their influence on neurons located in the ZI and on fibres traversing this structure [106, 114–118]. Therefore, as a next step, Milner and Mogenson [115] found that intraincertal microinjections of bicuculline (an antagonist of GABA_A_ receptors), picrotoxin (a blocker of GABA_A_ receptor channel) or glutamate increased locomotor activity of rats. These results suggested real contribution of the activated incertal neurons to this behaviour. In the same year, we published a study [119] which showed hyperlocomotion induced by bicuculline injected bilaterally into the region which we called “zona incerta-lateral hypothalamus (ZI-LH)” in rats. The region explored by us included medial part of the central region of the ZI, dorsal part of the hypothalamus and, at the most caudal level, rostral extension of Forel’s fields [120]. It partly overlain the area described by Mogenson and coworkers [106, 115, 116]. Hypermotility in rats was observed also by others as a result of intraincertal (into the rostral, central and/or caudal regions of the ZI) injections of picrotoxin and bicuculline, as well as agonists of AMPA/kainate receptors [121–123].

The first report suggesting a role of the ZI in akinesia and muscle rigidity came from our laboratory. In 1987, Wardas and coworkers [124] published a paper showing that picrotoxin or bicuculline injections into the ZI–LH region in rats reduced or even blocked the morphine-induced catalepsy and muscle rigidity (measured as a tonic electromyographic activity in the gastrocnemius muscle). Moreover, we found that bicuculline administered into this region at doses of ≥ 0.5 ng/0.5 μl/side, which were much lower than those necessary to obtain similar results after injections into the Vm (≥ 25 ng/0.5 μl/side), reduced the catalepsy induced by haloperidol [119]. On the other hand, muscimol which is an agonist of GABA_A_ receptors injected into this region evoked strong catalepsy which resembled that induced by haloperidol [119]. Since the haloperidol-induced catalepsy is a well-established rodent model of neuroleptic-induced parkinsonism in humans (mainly parkinsonian akinesia), the results of our study [119] were the first suggestion that ZI GABA synapses may be involved in parkinsonian signs independently of the Vm [119].

The neuroleptic-induced catalepsy is generally accepted to stem from blockade of dopamine D2 and D1 receptors localized in the striatum [125]. Therefore, in our following studies we tried to trace the route of impulse flow involved in this phenomenon from the striatum to the ZI. We found that catalepsy induced by intrastriatal injections of antagonists of D1 (SCH 23390) and D2 (sulpiride) receptors, or intrapallidal (into GPe) injections of muscimol was inhibited or even blocked by low doses of bicuculline administered into the ZI–LH region [126, 127]. Furthermore, the catalepsy induced by intrastriatal injections of sulpiride was inhibited by bicuculline administered into the GPe and muscimol into the SNr [127]. Finally, catalepsy induced by muscimol injected into the GPe was inhibited by muscimol injected to the SNr [127]. According to the above results, we concluded that ZI–LH was a link in a neuronal chain transmitting impulses pertinent to the neuroleptic-induced parkinsonism which were conveyed from the striatum to this region successively via GPe and SNr.

A role of the ZI region in transmission of neuronal impulses arising from the striatum was suggested also by Supko and coworkers [123] who found that the stereotypy induced by apomorphine or amphetamine was inhibited by the ibotenic acid-induced lesion or by a blockade of AMPA/kainate receptors of the central region of the ZI. On the other hand, the apomorphine- and amphetamine-induced stereotypy was also ameliorated by activation of AMPA receptors in the caudal portion of this structure [128].

Further support for a potential role of the ZI neurons in Parkinson’s disease came from the study of Périer and coworkers [129]. These authors found that a unilateral lesion of the nigrostriatal pathway increased both the level of mRNA coding for cytochrome oxidase subunit I [(COI), a metabolic marker of neuronal activity], and firing rate of neurons in the dorsal and lateral regions of the central ZI. In general, this region was located more caudally and more laterally to the region explored by us [119, 124, 126, 127]. In contrast, such lesion did not increase the number of Fos-immunoreactive cells in this region [130]. However, Heise and Mitrofanis [131] found that a unilateral lesion of the nigrostriatal tract resulted in a dramatic loss of parvalbumin expression in the neurons of central ZI (mainly in the ventral sector of this structure). These neurons are GABAergic [72, 75], and Heise and Mitrofanis [131] suggested that the decrease in parvalbumin (which is a calcium buffer protein) led to an increase in their excitability.

## Potential mechanisms involved in therapeutic effects of DBS of the ZI

The mechanisms underlying the therapeutic effect of DBS delivered to different structures are unclear at present and may depend on the stimulated region. DBS is an unspecific procedure which may stimulate orthodromically and antidromically myelinated axons passing through a given region, may influence terminals of inhibitory and excitatory efferents and antidromically their sources, may affect neuronal cell bodies or even non-neuronal cells [6, 132]. Contribution of each of these elements to the therapeutic efficiency of DBS of the ZI has not been analyzed, yet. However, a huge effort has been made to clarify the mechanisms involved in DBS of other structures, e.g. the GPi, STN or Vim in humans or parkinsonian primates and rodents [7, 132, 133]. Since therapeutic effects of DBS of these structures in PD patients appeared to be similar to those induced by their lesions, it has been initially suggested that DBS induces “a reversible (functional) lesion”. However, because of conflicting results with regard to inhibitory vs*.* stimulatory influences of DBS on output axons of the stimulated nuclei [7, 132, 133], and DBS-induced de-coupling of somatic and axonal firing [7], therapeutic effectiveness of this procedure has recently been suggested to depend on interrupting a pathological pattern of neuronal firing in the stimulated nucleus and disrupting abnormal information flow through the cortico-basal ganglia-cortical loop [7, 9].

As mentioned above, the ZI is traversed by fibres of pallidothalamic, nigrothalamic and cerebellothalamic projections [87, 88, 90, 100] which convey pathological impulses involved in generation of bradykinesia, muscle rigidity and various forms of tremor [6, 11–14, 16]. Therefore, it cannot be excluded that stimulation of these fibres by DBS is the main cause of amelioration of these signs. However, animal (rodent) studies have shown that pharmacological manipulations within this structure influenced motor behaviour and induced or counteracted parkinsonian-like disturbances [115, 119, 121–124, 126–128]. Therefore, these animal studies clearly suggested that, besides fibres passing through, neurons of the ZI may also be important for the generation of parkinsonian signs.

Is firing pattern of the ZI neurons pathological in PD? It has been shown that background neuronal activity of the ZI recorded in PD patients during stereotaxic operation differs significantly from that of neighbouring STN with respect to its low amplitude and a lack of proprioceptive responses [22, 134]. Signal analysis showed heterogeneous firing patterns, ranging from tonic to burst and paused units [134]. Since there are no data available regarding the ZI neuronal activity in normal, healthy humans, it is difficult to conclude to what extent that observed in PD patients is pathological. However, bursting of some of ZI neurons (3.4% of total) was synchronized with tremor at rest which could indicate a certain role of neurons of this structure at least in this symptom [134]. Moreover, the aforementioned studies [129, 131] suggested an increase in both firing of the ZI GABAergic neurons and their metabolic activity in 6-hydroxydopamine-induced rodent model of PD.

However, behavioural experiments in rodents seem to be in contradiction to the concept that activation of the ZI neurons underlies parkinsonian signs. As mentioned above, these studies showed that intraincertal injections of antagonists of GABA_A_ receptors or agonists of glutamate receptors, carried out along the whole extension (rostral–caudal) of the ZI, increased locomotor activity of rats [115, 119, 121, 122, 128]. Moreover, catalepsy induced by neuroleptics was antagonized by the blockade of GABA_A_ receptors in the ZI, while stimulation of these receptors by their agonist, muscimol, induced catalepsy [119, 126, 127]. Since the stimulation of GABA_A_ receptors can be expected to inhibit neurons, while their blockade or stimulation of glutamate receptors—to disinhibit or activate them, the above results may suggest that decreased, rather than increased, firing of some (undefined) ZI neurons is responsible for parkinsonian-like behaviour, at least in these models.

**Fig. 4:**
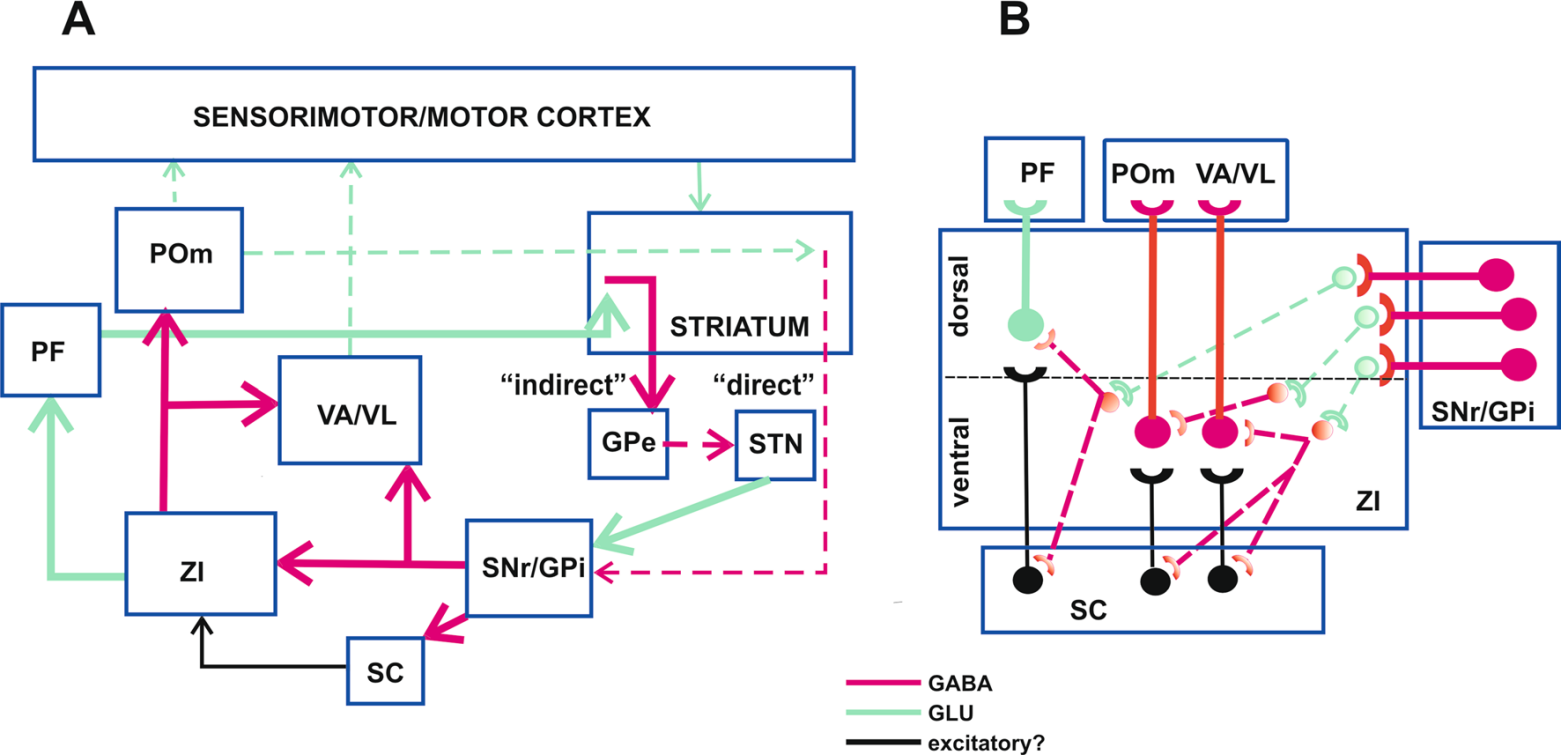
**a** The zona incerta (ZI) as a link in a neuronal chain responsible for conveying impulses potentially involved in motor signs of Parkinson’s disease. Due to dopaminergic deficiency in PD and imbalance between the “indirect” and “direct” GABAergic pathways arising from the striatum, the basal ganglia GABAergic output projections from the SNr/GPi to the VA/VL thalamic nuclei are overactive which results in inhibition of thalamic neurons and their projections to the sensorimotor/motor cortex [13]. The ZI receives GABAergic input from the SNr/GPi [71, 79, 87, 91] which is probably overactive, as well. The ZI, in turn, sends glutamatergic efferents to the PF [68, 77, 92] and GABAergic ones to the POm and VA/VL [68, 76, 77, 82] which are under excitatory influence of pathways originating from the SC [77], which also receives the GABAergic input from the SNr/GPi [15, 86]. Since glutamatergic pathways from the PF and POm to the striatum activate the “indirect” and “direct” pathways, respectively [77, 135, 136, 139], it has been suggested that the ZI, via inhibition of the POm, may suppress the “direct” pathway, while, via activation of the PF, may sensitize the “indirect” pathway to excitatory input from the sensorimotor/motor cortex [77, 136]. It may be speculated, therefore, that if both these incerto-thalamic pathways are overactive in PD they may contribute to the imbalance between the “direct” and “indirect” pathways and in this way, may be involved in appearance of motor signs of this disease. Moreover, putative activation of the GABAergic incerto-thalamic projection, which terminates in the VA/VL may constitute an additional, to the basal ganglia, source of inhibition of these nuclei in PD. **b** A hypothesis of potential, internal mechanisms of the ZI involved in modulation of incoming signals from the basal ganglia (SNr/GPi) and their transmission to thalamic nuclei (PF, POm, VA/VL) in PD. The ZI possesses extensive internal incerto-incertal network: GABAergic interneurons [107], collaterals of GABAergic incertal projection neurons [76, 99], and incerto-incertal connections linking different ZI regions utilizing undefined neurotransmitters [68, 108]. GABAergic incertal neurons are localized mainly in the ventral sector of this structure [66, 72, 77, 99], whereas glutamatergic ones concentrate mainly in its dorsal subdivision [66, 71, 72]. The GABAergic nigro(pallido)-incertal pathway (arising from the SNr/GPi) terminates mainly in the dorsal sector of the ZI [71, 79, 91], where it may switch to and inhibit glutamatergic neurons sending their axons to the ventral sector. A decrease in glutamatergic transmission in the ventral ZI may lead to inhibition of some GABAergic neurons terminating on incerto-thalamic neurons in the dorsal and ventral ZI, projecting to the PF, and POm/VA/VL, respectively [68, 76, 77, 92]. Inhibition of the internal GABAergic network may, in turn, lead to disinhibition of incerto-thalamic pathways, which are additionally under excitatory influence of signals coming from the SC [77]. Therefore, due to the above complex incerto-incertal interactions, overactivation of inhibitory basal ganglia input to the ZI, may increase output of this structure to the thalamus. Additionally, GABAergic incerto-collicular pathway starting in the ventral ZI [78–84] may be inhibited indirectly by the nigro(pallido)-incertal pathway that may result in disinhibition of the colliculo-incertal pathway and potentiation of its excitatory influence on incertal outputs to the thalamus. Brain structures: *GPe* external segment of the globus pallidus, *GPi* internal segment of the globus pallidus, *PF* parafascicular nucleus, *POm* posteromedial thalamic nucleus, *SC* superior colliculus, *SNr* substantia nigra, pars reticulata, *STN* subthalamic nucleus, *VA/VL* ventroanterior/ventrolateral nuclei of the thalamus, *ZI* zona incerta. Neuronal pathways: *GABA* GABAergic pathways, *GLU* glutamatergic pathways, excitatory?—putative excitatory pathways. Bold neurons and arrows—indicate activated neurons, pale neurons and dashed arrows—indicate inhibited neurons

Anatomical data presented above indicate that the ZI is a big communication node in neuronal network involved in the generation of parkinsonian signs (Figs. 3, 4). This region is reciprocally connected with the basal ganglia, cerebellum, several thalamic nuclei, different cortical regions, brainstem nuclei, and spinal cord (see above) (Fig. 3). Therefore, the ZI not only receives impulses from these structures but may retrogradely modulate their function, as well. This creates the basis for therapeutic action of DBS of the ZI which may disrupt pathological impulses transmitted through this structure. Although all the above connections may be involved both in PD and DBS effects, available functional data allow for discussion of the role of at least some of them (Fig. 4). First, the ZI receives projections from output structures of the basal ganglia—the SNr and GPi (Figs. 3, 4) [71, 79, 87, 91]. These pathways are formed at least partly by fibres of GABAergic pallidothalamic and nigrothalamic pathways which on their way to ventral and intralaminar thalamic nuclei make synaptic contacts in the ZI [87, 91]. Since according to the present knowledge these pathways are activated in PD and strongly inhibit their targets in the thalamus [6, 11–14], they possibly influence neurons of the ZI in the same way (Fig. 4b). Our results showing cataleptic effect of intraincertal muscimol seem to support this view and may suggest that this compound mimicked hyperactive GABAergic input to this structure [119].

What processes inside the ZI may be involved in reversing neuronal inhibition induced by the basal ganglia inputs [119] to neuronal excitation described in models of parkinsonism [129, 131]? In fact, intrinsic neuronal arrangement of the ZI is very complex. The ZI projection (principal) neurons seem to be under influence not only of afferents entering this structure but also of a network of inhibitory interneurons [107], as well as recurrent collaterals of inhibitory efferents [76, 99], and/or intranuclear incerto-incertal fibres, which connect different regions of this structure ipsi- and contralaterally [68, 108] (Fig. 4b). In this way, inhibition of neurons located at the site of input from the basal ganglia may lead to their own feedback disinhibition and inhibition or disinhibition of other neurons. Finally, flow of impulses in efferent pathways of the ZI depends on interactions between afferents of this structure and its entangled intrinsic network (Fig. 4b). In an agreement with complexity of ZI GABAergic neuronal network, Moon et al. [112] and Moon and Park [113] reported that both muscimol and bicuculline injected into the central region of this structure increased firing rate of some recorded neurons.

As mentioned above, the ZI sends intensive projections to intralaminar, associative and to a lesser degree also to ventral thalamic nuclei [68, 76, 77, 82–84, 92] (Fig. 3). At least some of these connections may be involved in parkinsonian signs.

First, incerto-thalamic pathway which terminates in VA/VL nuclei is, the most probably, GABAergic [76] and forms an additional, to the basal ganglia, source of their inhibition. Therefore, activation of this pathway (if present) may work concomitantly with basal ganglia-thalamic fibres to shut down neuronal activity in these thalamic regions in PD (Fig. 4).

Recently, a new concept has arisen with respect to the role of the ZI in regulation of thalamic gating of information flow from the cortex to the dorsolateral striatum [77, 135, 136]. The dorsolateral striatum, which is involved in the execution of habitual behaviours in a familiar sensory context, receives, besides the major corticostriatal input, also excitatory projections: from intralaminar—PF nucleus and from the higher order—posteromedial thalamic nucleus (a rodent equivalent of primate anterior pulvinar nucleus; POm [85, 135, 137–139]). These two thalamic projections influence responses of the striatal medium-spiny neurons to cortical signalling in an opposite way [135, 139]. Fibres arising from the PF nucleus activate burst-pause pattern of spiking of striatal cholinergic interneurons which initially, transiently interrupts, and then enhances responsiveness of striopallidal GABAergic neurons to corticostriatal input [135, 139]. The latter mechanism is probably important for redirection of attention and interruption of ongoing motor activity with the presentation of salient stimulus [135, 139]. On the other hand, the thalamostriatal projection from the POm activates the strionigral GABAergic pathway, and secondarily inhibits neurons in the SN [136], which leads to facilitation of movements [136]. The PF nucleus receives a projection from the dorsal sector of the ZI which is probably glutamatergic [68, 77, 92, 135], while POm is supplied by GABAergic fibres from the ventral subdivision of the ZI [68, 76, 77, 135] (Fig. 4). These anatomical data indicate that the ZI may suppress strionigral neurons indirectly by inhibition of the POm, and sensitize striopallidal neurons by activation of the PF. It may be speculated, therefore, that if both these incerto-thalamic pathways are overactive in PD they may enhance the imbalance between the “direct” and “indirect” pathways in PD and in this way, may contribute to appearance of motor signs of PD (Fig. 4).

On the other hand, inhibition of the ZI by DBS may restore normal functioning of striatal output neurons which may result in therapeutic effect of this procedure. In an agreement with this concept, Benazzouz et al. [140] found that DBS of the ventral sheet of the ZI reversed metabolic effects of the lesion of dopaminergic neurons in rats. While the lesion increased COI mRNA expression in the substantia nigra pars reticulata, and decreased it in the GPe, the DBS of the ZI had an opposite effect. Interestingly, this effect was similar to that of DBS of the STN [140].

Our hypothesis concerning contribution of the ZI and its internal network to a neuronal circuit responsible for motor signs of PD is shown in the Fig. 4a, b. This hypothesis is based on the assumption that an increased GABAergic inhibitory input to the ZI may diminish activity of incerto-incertal connections (glutamatergic and GABAergic), and in this way, may disinhibit the above incerto-thalamic pathways, which secondarily, via thalamostriatal projections, may disturb activity of striatal outputs. Future research in animals is warranted to verify this hypothesis. The first step may possibly consist in establishing how stimulation (optical?) of the nigral and pallidal GABAergic output pathways will influence activity of different, electrophysiologically and neurochemically identified populations of incertal neurons, and neurons of their thalamic targets.

As mentioned above, the ZI conveys also information arising from the nucleus accumbens and subpallidal region (substantia innominata) to the MLR (Fig. 3). This neuronal system has been suggested to be involved in initiation of exploratory locomotor activity [106, 116, 141, 142]. The MLR, which contains nuclei interconnected with the ZI (PPN, cuneiform nucleus), is a coordination centre for activation and control of spinal locomotor generator neurons [118, 141, 143]. The MLR is also connected with the basal ganglia, thalamus and other structures suggested to be involved in PD pathology [11, 144], and neurons of the PPN has been found to be inhibited in animal models of PD [145, 146]. What is interesting, the ZI is interconnected mainly with pars dissipata of the PPN, a subregion, which receives projections from the GPi and SNr, and contains bursting neurons (probably glutamatergic) involved in initiation of programmed movements [5]. In an agreement with the physiological function of the MLR, the DBS of PPN improves mainly postural instability and refractory gait freezing in PD [5, 8], and simultaneous stimulation of the PPN and caudal ZI [147, 148], PPN and STN [149] or PPN and GPi [8] has been used in PD patients to achieve an optimal therapeutic effect.

The last issue which should be mentioned here is that despite differential neuroarchitecture of the ZI and multiplicity of its connections DBS induces antiparkinsonian effects when applied either into the rostral/central or caudal region of this structure. Although, the reason of this phenomenon is not clear at present, the existence of extensive intrinsic network of the ZI [68, 107, 108] may explain it, at least partly. Thanks to incerto-incertal connections, the whole ZI is in a position to integrate signals coming from functionally diverse brain centres to its different sectors. Furthermore, neurons of one sector of the ZI may influence impulses leaving other sectors both ipsi- and contralaterally [108].

However, according to general opinion of neurosurgeons, DBS of the caudal sector of the ZI was the most therapeutically effective, especially with respect to reduction of different forms of tremor, including parkinsonian tremor [46, 47, 49–53]. The most probable explanation of this effect is that DBS affects the cerebellothalamic glutamatergic fibres arising from subcortical deep cerebellar nuclei [150] which traverse this region and form there collaterals and terminals [88, 90, 100].

## Concluding remarks

The analysis of the available clinical and anatomical data, as well as results obtained in animal models of parkinsonism allows for drawing the following conclusions.

The ZI, which has interconnections with most of structures involved in PD pathology, is an important target for DBS, which alleviates parkinsonian bradykinesia, muscle rigidity and tremor. The therapeutic effect of DBS of the ZI may be related to disruption of abnormal information flow through this structure due to its influence on intrinsic incertal network and projection neurons, as well as fibres en passage (e.g. pallidothalamic and cerebellothalamic). The cerebellothalamic fibres traversing the caudal sector of the ZI, as well as their collaterals terminating in this region may contribute significantly to the tremorolytic efficiency of the DBS of this part of the ZI.

However, since the ZI is located close to the STN, the current spread to the STN during each session of ZI DBS, cannot be excluded. Therefore, the challenge for future research lies in discrimination of contribution of each structure to therapeutic effects of DBS. Studies on the effects of DBS of the ZI vs*.* STN in animal models of PD on activity of different neuronal populations in structures belonging to the PD-related circuits would be of help to solve this problem.
